# Impaired resolution of DSS-induced colitis in mice lacking the glucocorticoid receptor in myeloid cells

**DOI:** 10.1371/journal.pone.0190846

**Published:** 2018-01-11

**Authors:** Garrit K. Meers, Hanibal Bohnenberger, Holger M. Reichardt, Fred Lühder, Sybille D. Reichardt

**Affiliations:** 1 Institute for Cellular and Molecular Immunology, University Medical Center Goettingen, Göttingen, Germany; 2 Institute of Pathology, University Medical Center Goettingen, Göttingen, Germany; 3 Institute for Multiple Sclerosis Research and Neuroimmunology, University Medical Center Goettingen, Göttingen, Germany; Duke University, UNITED STATES

## Abstract

Inflammatory bowel disease (IBD) is a highly prevalent intestinal disorder for which no cure exists. Currently, the standard first-line treatment of IBD consists of systemic glucocorticoid (GC) application, even though therapy can be complicated by unresponsiveness or adverse effects. In view of the importance of macrophages and neutrophils for the pathogenesis of IBD we set out to define the relevance of these cell types as targets of GC using the mouse model of DSS-induced colitis. We found that the disease did not resolve in GR^lysM^ mice lacking the GC receptor (GR) in myeloid cells after removal of the chemical insult. While clinical symptoms and tissue damage in the colon ameliorated again in GR^flox^ mice, the disease further aggravated in GR^lysM^ littermates. The observed difference coincided with an increased abundance of macrophages in inflammatory infiltrates in the colon of mutant mice whereas neutrophil and T cell numbers were similar. Concomitantly, systemic IL-6 secretion and mRNA levels of pro-inflammatory cytokines in the colon were elevated in GR^lysM^ mice and gene expression of scavenger receptors and IL-10 was diminished. Taken together, our results reveal an important role of myeloid cells as targets of GC in DSS-induced colitis and probably in IBD in humans as well.

## Introduction

The gastrointestinal (GI) tract is constantly exposed to a variety of commensal as well as potentially harmful microbiota and therefore requires a well-balanced control of the local immune system [1]. When intestinal homeostasis is disturbed, pathogenic immune responses arise that are commonly known as IBD [2]. This syndrome comprises two types of intestinal disorders designated ulcerative colitis (UC) and Crohn´s disease (CD), both of which represent major health problems in Western societies with an overall prevalence of about 200 cases per 100.000 persons. Whereas inflammation in UC is restricted to the mucosal layer of the colon, CD can affect the entire intestine. The onset of IBD mostly occurs in early adulthood and is accompanied by symptoms such as abdominal pain, weight loss, diarrhea and rectal bleeding [3]. Risk factors for the development of IBD include genetic and environmental aspects [4]. On the one hand, twin studies and the high incidence of IBD among close relatives point towards a genetic contribution to disease susceptibility, and indeed a number of genes associated with IBD have been identified in recent years. On the other hand, the higher risk of IBD in persons who adopt a westernized lifestyle with a high fat and sugar diet, smoking, stress and the regular use of antibiotics clearly supports a contribution of environmental aspects to this disease.

Besides surgical treatment such as colectomy, which in itself carries a high level of affliction for the patients, there are a number of newly developed therapeutics available for the treatment of IBD, in particular monoclonal antibodies targeting TNF-α and integrin α4 [5]. While several clinical trials have provided promising results, application of these biologicals was also accompanied by adverse effects, including infusion reactions, infection-related mortality, malignancy and autoimmunity. Despite the progress made with these new agents, GC application remains the gold standard for the treatment of IBD [6]. Prednisolone is the preferred GC in the clinic and generally administered at a dose of 40 mg/day per os. About 40% of the patients respond well to GC therapy and show a rapid amelioration of clinical symptoms, allowing for tapering of the dose and eventually discontinuation of the treatment. In contrast, there are patients referred to as being steroid-dependent who require prolonged treatment since they relapse after tapering. Finally, a further 15–20% of the patients are GC-resistant. Since GC can cause extensive adverse effects such as osteonecrosis, infection, hyperglycemia, and gastric ulcer, the duration of GC treatment is usually kept as short as possible and at best limited to the management of acute inflammation [6]. However, long-term application may become necessary in patients with refractory disease, and under such circumstances, GC therapy often has to be stopped due to adverse effects and second line therapy imitated. As an alternative, new strategies to prevent side effects of GC are currently being developed including the targeted delivery by nanocarrier systems [7]. For example, the application of a GC-copolymer conjugate has been found to ameliorate disease symptoms in a mouse model of IBD while preventing the development of osteoporosis [8, 9]. Considering the central role of GC in IBD therapy and the complications associated with their use, further efforts aimed at optimizing this therapy are warranted.

The pathomechanism of IBD involves a complex interplay between structural cells of the GI tract and various types of resident and infiltrating immune cells [1]. Whereas the intestinal epithelium provides a physical barrier against invading microorganisms, innate immune cells such as dendritic cells and macrophages located in the connective tissue beneath the epithelium form a first line of defense against potentially harmful bacteria. Among these cells, macrophages have been found to play a crucial role in the maintenance of gut homeostasis [10]. A suitable model with which to analyze the function of the innate immune system in particular for the development of UC is the treatment of mice with dextran sodium sulphate (DSS), a chemical compound that damages the colonic mucosa, resulting in an inflammatory response mainly mediated by macrophages and neutrophils. It is noteworthy that activated macrophages can adopt two distinct phenotypes, both of which play important roles in the pathogenesis of IBD. M1 cells secrete pro-inflammatory cytokines and mediators whereas M2 cells rather express anti-inflammatory cytokines and scavenger receptors involved in immunoregulation and tissue repair [11]. Using the DSS-induced colitis model it could be shown that intestinal inflammation is strongly influenced by endogenous GC, which exert prominent effects on myeloid cells after being released from the adrenal gland or locally generated in the gut [12]. Especially the synthesis of GC in the intestine *in situ* was found to be important for the regulation of intestinal inflammation and to be differently regulated than adrenal synthesis [13, 14]. Nevertheless, the actual mechanisms by which GC from distinct sources control inflammation in the gut remain poorly understood.

The majority of GC effects are mediated by the GR, a member of the nuclear receptor superfamily, which is ubiquitously expressed in most cell types. In immune cells such as macrophages and neutrophils, GC inhibit the expression and secretion of pro-inflammatory cytokines and other mediators that are typical for M1 polarization [15]. Consequently, mice carrying a deletion of the GR in myeloid cells are prone to sepsis and acute lung injury [16, 17], highlighting the importance of the anti-inflammatory activity of GC in these cell types. In addition, GC induce the expression of anti-inflammatory cytokines and scavenger receptors typical for M2 polarization, which contributes to the resolution of inflammatory responses [18]. Considering the pathomechanism of IBD, we wondered which role these GC effects played in the modulation of DSS-induced colitis. Our findings revealed that a lack of the GR in myeloid cells impairs disease resolution as characterized by persisting clinical symptoms and tissue damage, presumably due to a deficit in controlling leukocyte infiltration into the colon, systemic cytokine release and local gene expression by immune cells in the lamina propria.

## Material and methods

### Animal experimentation

GR^flox^ (Nr3c1^tm2GSc^) and GR^lysM^ (Nr3c1^tm2GSc^Lyz2^tm1(Cre)Ifo^) mice on a C57BL/6 background have been described previously [19]. The mice were kept in individually ventilated cages under specific-pathogen-free conditions in our animal facility at the University Medical Center Göttingen and used at an age of 8–12 weeks. All animal experiments were conducted according to national and international guidelines (S1 Checklist) and approved by the responsible authority (*Niedersächsisches Landesamt für Verbraucherschutz und Lebensmittelsicherheit; Az*: *33*.*9-42502-04-12/0937)*.

### Induction of acute DSS-induced colitis

The induction of the disease was performed essentially as described by Wirtz and colleagues [20]. DSS (Sigma-Aldrich, Taufkirchen, Germany) was added to the drinking water at a concentration of 2% for 8 days. Thereafter, mice received normal tap water for another 2 or 4 days. The DSS solution was prepared freshly each time and changed every other day. Untreated controls received tap water only.

### Disease assessment

To evaluate disease severity, mice were daily weighed and evaluated for clinical symptoms. The disease activity index (DAI) was determined on a scale from 0 to 10 and calculated as the sum of three individual scores [21]. Weight loss was scored from 0 to 4 (0 = 0–1%, 1 = 1–5%, 2 = 5–10%, 3 = 10–15%, 4 = >15%). Individual stool samples were collected and evaluated for consistency, resulting in a score of 0 to 2 (0 = normal, 1 = soft, 2 = diarrhea). Intestinal bleeding was judged with the help of a fecal occult blood test (Hemocare, Care Diagnostica, Voerde, Germany) and scored from 0 to 4 (0 = negative hemoccult, 1 = green hemoccult, 2 = blue hemoccult, 3 = blood visible, 4 = rectal bleeding). Animals that died or had to be sacrificed for ethical reasons were assigned a score of 12.

### Histology

After sacrifice, the colon was removed and its length measured. The colon was flushed with ice-cold PBS, cut open longitudinally and rolled up from distal to proximal to obtain a so-called “swiss-role” [22], which was then fixed in 4% PFA (Carl Roth, Karlsruhe, Germany) overnight at room temperature. After dehydration and embedding in paraffin, 2 μm sections were prepared and stained with hematoxylin and eosin (H&E). Photomicrographs were acquired using a Leica Axio Scope A1 microscope (Wetzlar, Germany). Histopathological evaluation of the entire section was performed in a blinded manner by determining crypt distortion, inflammation and loss of goblet cells. Based on these criteria an inflammatory score from 0 to 3 (0 = none, 1 = mild, 2 = moderate, 3 = severe) was assigned, multiplied with the percentage of the affected tissue area, and depicted on a scale from 0 to 10.

### Immunohistochemistry

For immunohistochemical stainings, 2 μm tissue sections were incubated in EnVision Flex Target Retrival Solution (Low or High pH; Dako, Santa Clara, CA), followed by incubation with primary antibodies recognizing CD68 (1:200; Abcam, Cambridge, UK), CD3 (1:2000; Santa Cruz Biotechnology, Heidelberg, Germany), GR1 (1:200; BD Biosciences, Heidelberg, Germany), or the GR (1:200; Santa Cruz Biotechnology) for 30 minutes at room temperature. Polymeric secondary antibodies coupled to HRP (ImmPRESS HRP Polymer Detection Kit; Vector Laboratories, Burlingame, CA) and DAB (Dako) were employed to visualize sites of immunoreactivity (dark brown staining). Hematoxylin was used for counterstaining and photomicrographs were acquired with a Leica Axio Scope A1 microscope.

### Flow cytometric analysis of lamina propria cells

Lamina propria cells were isolated from the colon essentially as described previously [23]. In brief, colons were removed, washed in PBS, opened longitudinally and incubated in PBS with 60 mM EDTA and 3 mM DTT (both from Carl Roth) on ice for 1 hour. Thereafter, colons were transferred into tubes containing PBS, and intestinal epithelial cells were removed by vigorous shaking for 1 min. The supernatant was discarded and the step was repeated two more times. Subsequently, the tissue was washed once with RPMI/10% FCS (ThermoFisher, Waltham, MA) and cut into small pieces. After addition of 100 U/ml collagenase 1A, 100 U/ml Collagenase II and 50 U/ml DNase II (all from Sigma) the colonic tissue was digested at 37°C for 30 min. During incubation, the tube was briefly vortexed every 3 to 5 min. Finally, the cell suspension was passed through a 40 μm cell strainer, washed once with PBS, counted with a Neubauer hemocytometer, and analyzed by flow cytometry. To this end, an FcR blockade was initially performed by incubation with TruStain fcX (anti-mouse CD16/32; clone: 93). After washing, the cell suspensions were stained with the following monoclonal antibodies (BioLegend, Uithoorn, The Netherlands): anti-CD3 (clone: 17A2), anti-CD4 (clone: RM4-5), anti-CD11b (clone: M1/70), anti-CD45.2 (clone: 104), and anti-LyG6 (clone: 1A8). Data were acquired on a FACS Canto II device (BD Bioscience) and analyzed using FlowJo software (Tree Star, Ashland, OR).

### ELISA

Serum was prepared from blood samples obtained by cardiac puncture. Levels of IL-6 were determined by ELISA using a commercially available kit according to the manufacturer’s instructions (BioLegend).

### GR^flox^ recombination analysis by PCR

Colon biopsies were digested with Proteinase K followed by DNA extraction according to standard protocols. The unrecombined GRflox (275 bp) and the recombined GRnull (390 bp) alleles were amplified as previously described [24], and the PCR fragments were separated on a 1.5% agarose gel.

### Quantitative RT-PCR

Pieces of frozen tissue were homogenized and total RNA extracted with the help of the RNeasy plus Universal Kit (Qiagen, Hilden, Germany). To prevent inhibition of the reverse transcriptase by residual DSS present in the RNA preparation [25, 26], mRNA was purified using the Dynabeads mRNA DIRECT Kit (ThermoFisher). Thereafter, the mRNA was reverse transcribed with a iScript Reaction Mix kit (Bio-Rad, Munich, Germany) followed by quantitative RT-PCR using the Power SYBR Mix (ThermoFisher). Results were normalized to the expression of the house-keeping gene HPRT and evaluated using the ΔΔCt method. In order to correct the mRNA levels of each gene for differences in macrophage numbers in the colon, the obtained values were finally normalized to F4/80 gene expression.

### Statistical analysis

Data was analyzed using GraphPad Prism software (San Diego, California, USA). Data is depicted as mean values ± SEM. The unpaired two-tailed Student’s t test or the Newman-Keuls Multiple Comparison test was used for statistical analysis. Levels of significance: *, p <0.05; **, p <0.01; and ***, p <0.001.

## Results

### DSS-induced colitis in mice lacking the GR in myeloid cells

**Fig 1:**
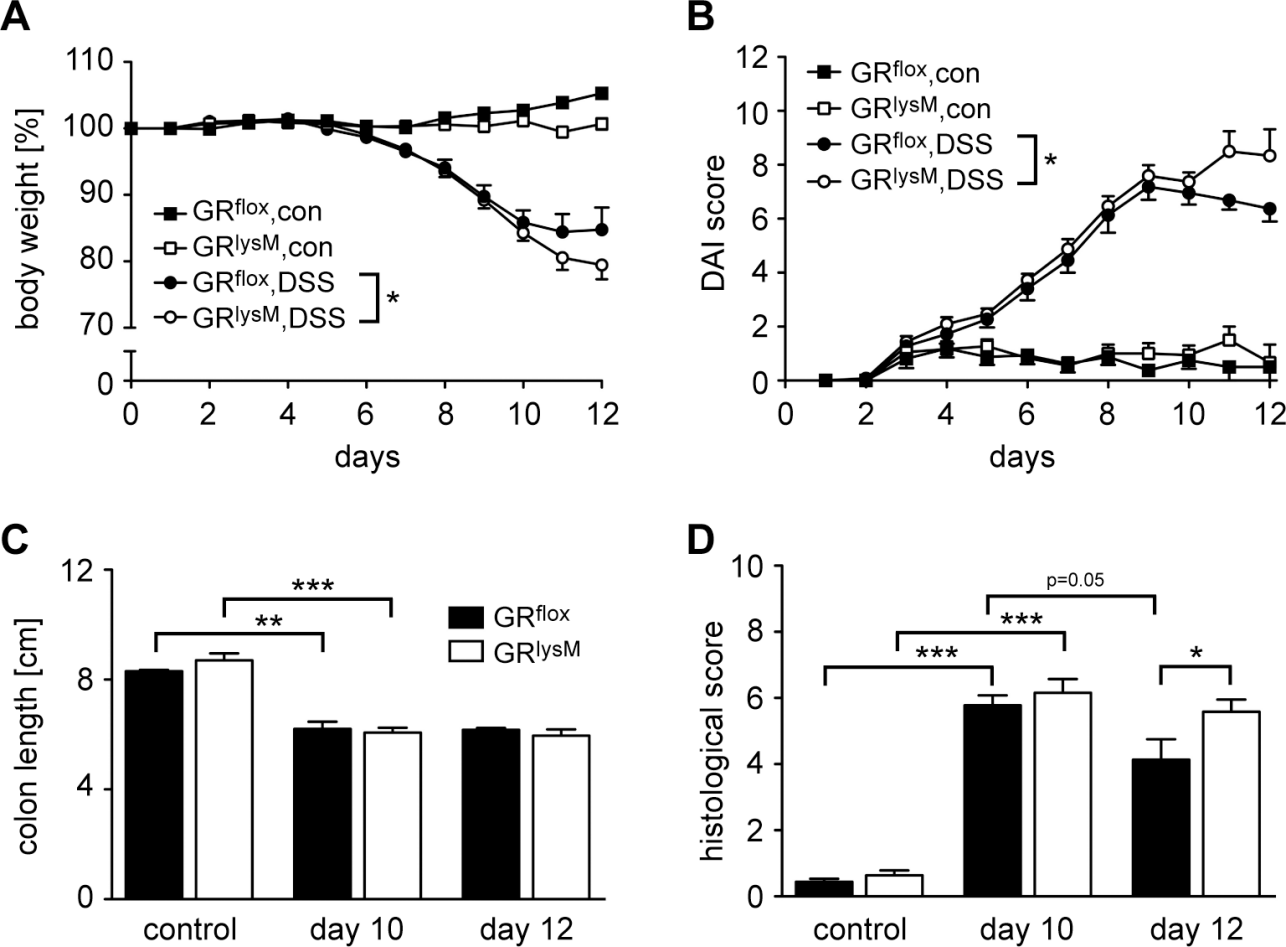
Features of DSS-induced colitis in GR^flox^ and GR^lysM^ mice. Mice were treated with 2% DSS in the drinking water for 8 days to induce the disease followed by a recovery phase lasting for another 4 days. Mice receiving tap water served as controls (con). (A) The body weight was determined daily for each mouse and is depicted as the percentage of the initial value measured before the onset of the experiment. (B) The disease activity index (DAI) comprising body weight, stool consistency and fecal blood was calculated daily and is depicted on a 12-point scale. GR^flox^ mice: N = 8/12 (con/DSS), GR^lysM^ mice: N = 9/16 (con/DSS). (C) Mice were sacrificed on days 10 or 12, the colons removed and their length measured from the cecal/colon junction to the rectum. GR^flox^ mice: N = 5/3/3 (con/10/12), GR^lysM^ mice: N = 6/7/5 (con/10/12). (D) Mice were sacrificed on days 10 or 12, the colons flushed, opened longitudinally and rolled up from distal to proximal to obtain a “swiss-role”. H&E stainings of histological sections were prepared and evaluated on the basis of crypt distortion, inflammatory infiltration, and loss of goblet cells. GR^flox^ mice: N = 9/6/8 (con/10/12), GR^lysM^ mice: N = 12/18/12 (con/10/12). All values are depicted as mean ± SEM. Statistical analysis was performed by unpaired two-tailed Student’s t test (*: p <0.05, **: p <0.01, ***: p <0.001). In panels A and B, statistical analysis was limited to the time span between days 11 and 12.

GR^lysM^ mice that specifically lack the GR in myeloid cells and their corresponding GR^flox^ littermates were treated with DSS for 8 days to induce inflammation in the colon. While untreated mice showed a constant body weight during the entire course of the experiment, mice receiving DSS strongly lost weight between days 7 and 10 regardless of their genotype (Fig 1A). GR^flox^ mice started to recover thereafter whereas GR^lysM^ mice continued to lose weight up to day 12 (Fig 1A). At the end of the experiment, weight loss differed significantly between the two genotypes. The monitoring of clinical symptoms confirmed our observation. The DAI score in DSS-treated mice of both genotypes rose until between day 9 and 10 and declined again thereafter in GR^flox^ mice (Fig 1B). In contrast, the DAI score in GR^lysM^ mice further increased over the last two days of the experiment (Fig 1B). Clinical symptoms were accompanied by a shortening of the colon on day 10, which did not reverse until day 12 in either genotype (Fig 1C). We conclude that the GR in myeloid cells is essential to achieve resolution of DSS-induced colitis.

### Histological assessment of colon inflammation

**Fig 2:**
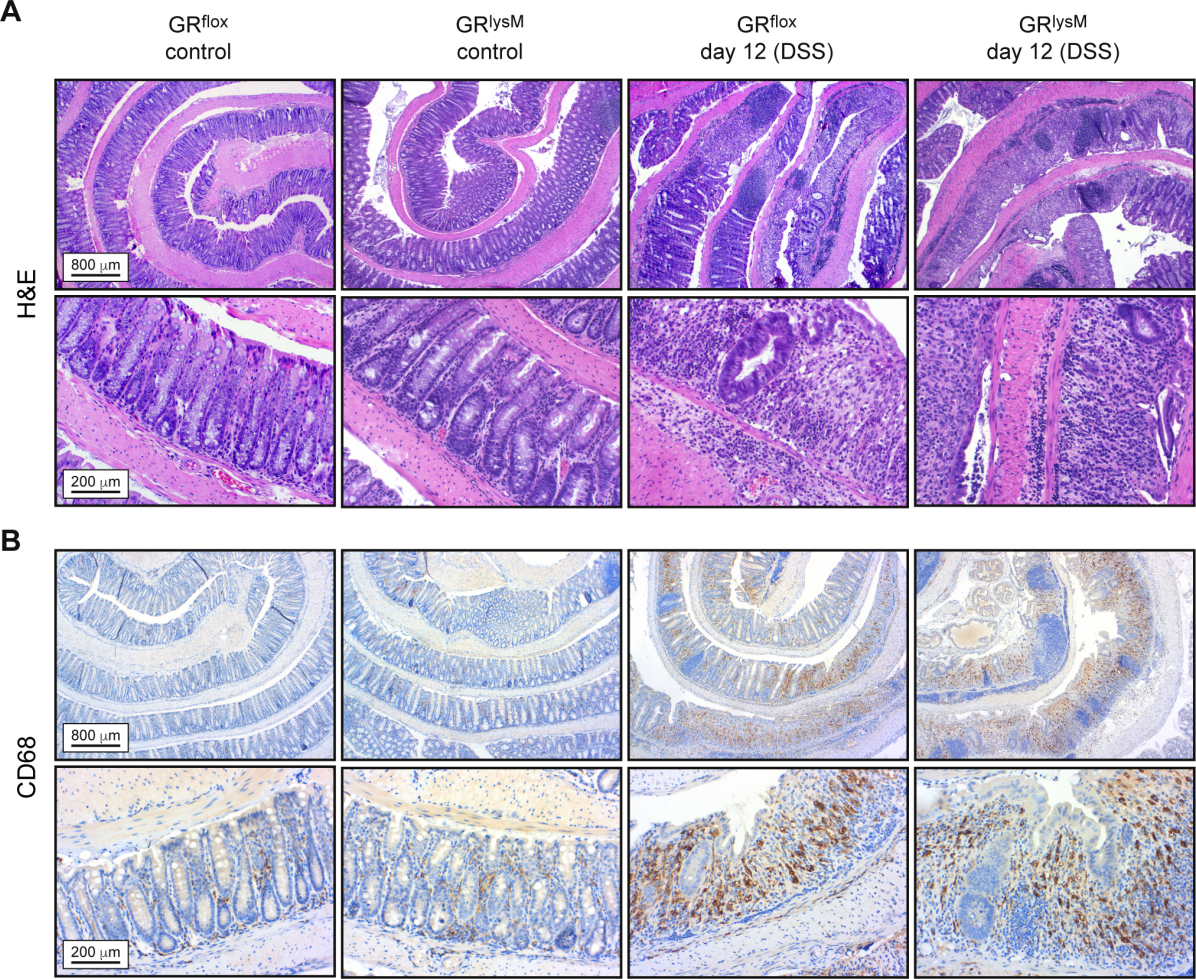
Histological and immunohistochemical stainings of colon sections prepared during the resolution phase of DSS-induced colitis in GR^flox^ and GR^lysM^ mice. Mice received 2% DSS in the drinking water for 8 days and were sacrificed on day 12. Mice receiving tap water served as controls. The colons were flushed, opened longitudinally and rolled up from distal to proximal to obtain a “swiss-role” for histological analysis. (A) Representative photomicrographs from H&E stained 2 μm colonic tissue sections at 5x (upper panel) or 20x (lower panel) magnification. (B) Representative photomicrographs from 2 μm colonic tissue sections incubated with an anti-CD68 antibody at 5x (upper panel) or 20x (lower panel) magnification. The higher magnification photomicrographs of DSS-treated mice are representative of heavily inflamed areas of the colon. Scale bars correspond to 800 μm and 200 μm, respectively.

A major hallmark of DSS-induced colitis is leukocyte infiltration into the colon wall resulting in massive tissue damage [27]. Hence we decided to evaluate histological alterations along the entire length of the colon (Fig 2A). Histopathological assessment of H&E stained tissue sections revealed a massive destruction of the epithelial lining, a shortening or absence of crypts, loss of goblet cells, and excessive leukocyte infiltrates in DSS-treated mice (Fig 2A). Generally, tissue damage was more pronounced in the distal part of the colon. Quantification of histopathological alterations on day 10 revealed similar scores in both genotypes, whereas tissue damage was significantly more severe in GR^lysM^ mice on day 12 compared to GR^flox^ mice (Fig 1D). Notably, the histological score in GR^flox^ mice improved during the last two days while it remained unaltered in GR^lysM^ mice. This finding is in support of an ongoing colonic inflammation in mutant mice, which is in line with the observed changes in body weight and DAI score (see Fig 1A and 1B). In summary, the failure of DSS-induced colitis to resolve in GR^lysM^ mice appears to be linked to persistent tissue damage in the colon.

### Characterization of the inflammatory infiltrate in the colon

**Fig 3:**
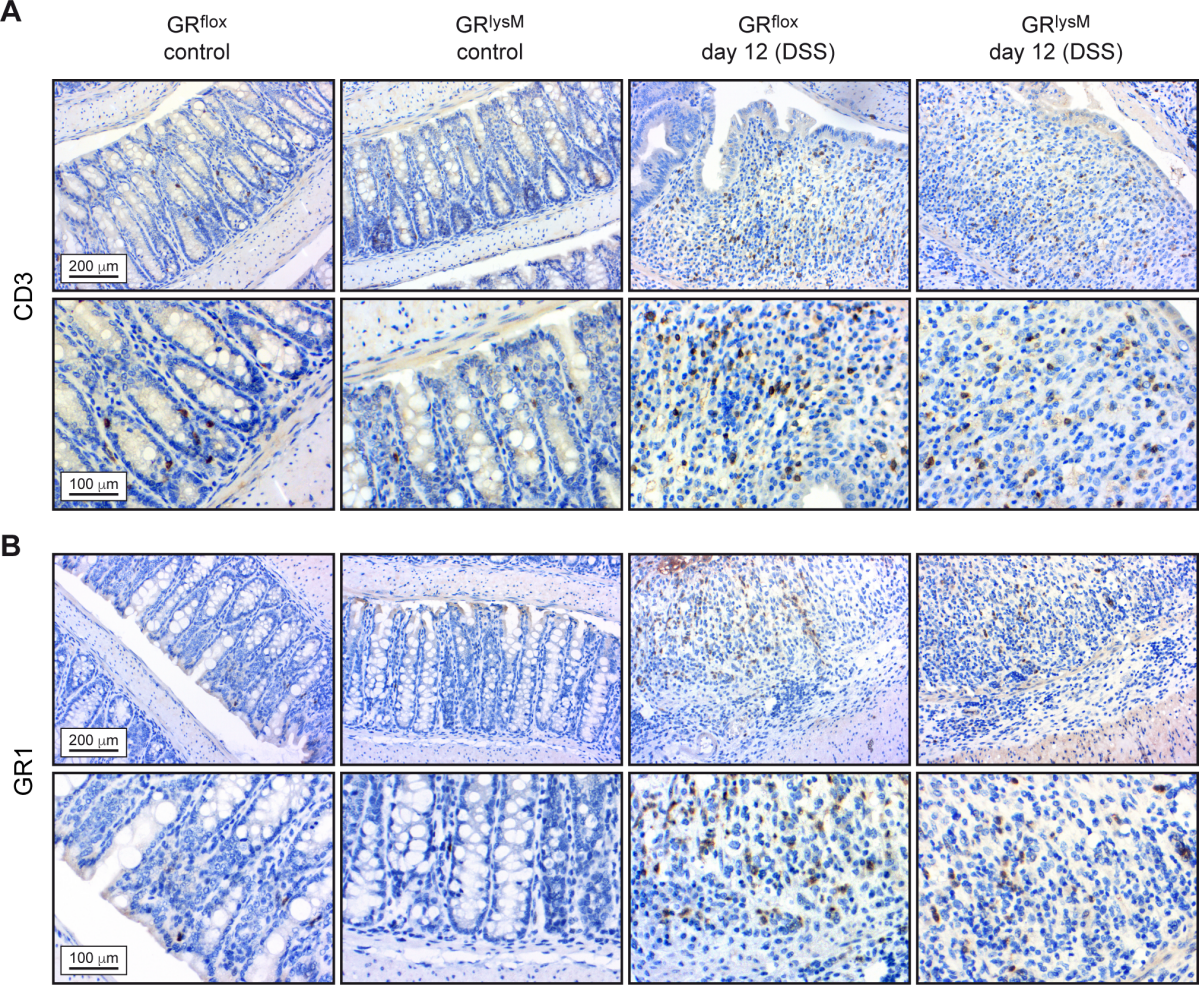
Immunohistochemical analysis of the colon during the resolution phase of DSS-induced colitis in GR^flox^ and GR^lysM^ mice. Mice received 2% DSS in the drinking water for 8 days and were sacrificed on day 12. Mice receiving tap water served as controls. The colons were flushed, opened longitudinally and rolled up from distal to proximal to obtain a “swiss-role” for histological analysis. Representative photomicrographs from 2 μm colonic tissue sections incubated with an (A) anti-CD3 or (B) anti-GR1 antibody at 20x (upper panel) or 40x (lower panel) magnification. Photomicrographs of DSS-treated mice in both panels are representative of heavily inflamed areas of the colon. Scale bars correspond to 200 μm and 100 μm, respectively.

**Fig 4:**
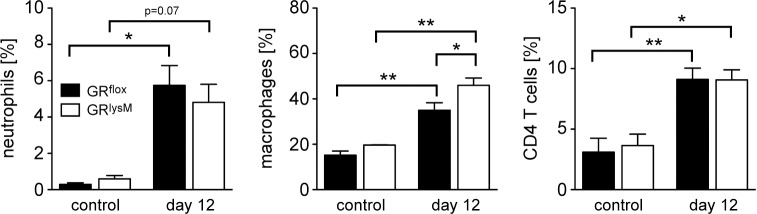
Flow cytometric analysis of lamina propria cells isolated from the colon during the resolution phase of DSS-induced colitis in GR^flox^ and GR^lysM^ mice. Mice received 2% DSS in the drinking water for 8 days and were sacrificed on day 12. Mice receiving tap water served as controls. Lamina propria (LP) cells were prepared from the colon by enzymatic digestion and subsequently analyzed by flow cytometry. The percentage of neutrophils and macrophages among LP cells was determined by staining with antibodies recognizing CD11b and Ly6G (left and middle panel), the percentage of CD4 T cells was determined by staining with antibodies recognizing CD3, CD4 and CD45 (right panel). GR^flox^ mice: N = 4/11 (con/12), GR^lysM^ mice: N = 2/8 (con/12). All values are depicted as mean ± SEM. Statistical analysis was performed by Newman-Keuls Multiple Comparison test (*: p <0.05, **: p <0.01).

To determine the composition of leukocytes present in the colon on day 12, we performed an immunohistochemical analysis. A considerable number of macrophages was already found in the lamina propria of untreated control mice whereas T cells and neutrophils were rare (Figs 2B, 3A and 3B). Infiltration of all three cell types was strongly increased in DSS-treated GR^flox^ and GR^lysM^ mice on day 12, but macrophages were also the most abundant leukocyte subset in the inflamed colon (Figs 2B, 3A and 3B). To independently confirm these results, we isolated cells from the lamina propria and characterized them by flow cytometry. In line with our previous analyses, colonic leukocytes in untreated GR^flox^ and GR^lysM^ control mice were mainly composed of macrophages, some T cells and a few neutrophils. Regardless of the genotype, a massive infiltration of these cells into the lamina propria was observed on day 12 after initiation of DSS treatment (Fig 4). Importantly, the numbers of neutrophils and CD4 T cells were similar in GR^flox^ and GR^lysM^ mice at this time point whereas macrophages were significantly more abundant in the colon of mutant mice on day 12 (Fig 4). Hence our data suggest that the extent of macrophage infiltration might impact the resolution of DSS-induced colitis.

### GR ablation in myeloid cells in GR^lysM^ mice

**Fig 5:**
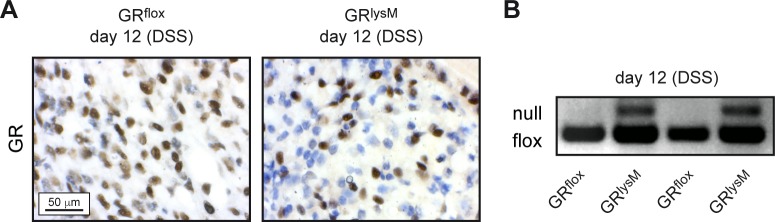
Presence of GR-deficient cells in the inflamed colon during DSS-induced colitis in GR^flox^ and GR^lysM^ mice. Mice received 2% DSS in the drinking water for 8 days and were sacrificed on day 12. (A) Representative photomicrographs from 2 μm colonic tissue sections incubated with an anti-GR antibody at 64x magnification. Comparable areas of severe inflammation are depicted for GR^flox^ and GR^lysM^ mice. The scale bar corresponds to 50 μm. (B) DNA was isolated from colonic tissue and the genomic region containing the loxP sites was amplified to detect recombination of the GR locus. PCR products were separated on an agarose gel. The analysis of two samples from each genotype is depicted. The “flox” band refers to the unrecombined GR locus, the “null” band refers to the recombined gene unable to encode an intact GR protein.

Previous studies revealed a high recombination efficacy of the GR gene in myeloid cells of GR^lysM^ mice, concerning about 70% of macrophages and 90% of neutrophils, whereas T cells were largely unaffected [28]. To confirm that the majority of infiltrating cells in mutant mice were devoid of the GR, an immunohistochemical analysis was performed. All cells in heavily infiltrated colonic areas in the colon of DSS-treated GR^flox^ mice on day 12 stained positive for the GR. Analysis of similar regions in GR^lysM^ mice, however, revealed the presence of many cells that lacked the GR (Fig 5A). PCR analysis confirmed this finding by demonstrating recombination of the GR gene in a subset of cells present in the colon of DSS-treated GR^lysM^ but not GR^flox^ mice (Fig 5B). Collectively, our findings support the notion that the phenotype of mutant mice is due to a deletion of the GR in colon infiltrating cells.

### Systemic cytokine secretion during DSS-induced colitis

**Fig 6:**
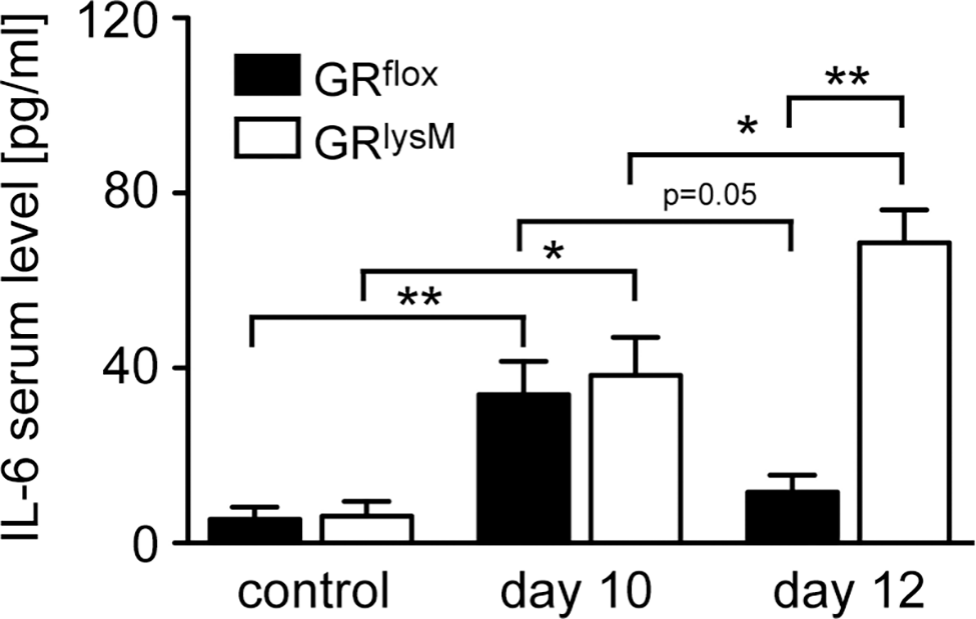
Systemic IL-6 secretion during DSS-induced colitis in GR^flox^ and GR^lysM^ mice. Mice received 2% DSS in the drinking water for 8 days and were sacrificed on day 10 or 12. Mice receiving tap water served as controls. Blood was collected through cardiac puncture and serum IL-6 levels were determined by ELISA. GR^flox^ mice: N = 5/3/4 (con/10/12), GR^lysM^ mice: N = 6/7/5 (con/10/12). Statistical analysis was performed by unpaired two-tailed Student’s t test (*: p <0.05, **: p <0.01).

Previous observations in a model of sepsis indicated that GR^lysM^ mice suffer from an inability to control systemic cytokine secretion [16]. We therefore studied levels of pro-inflammatory cytokines in the serum on days 10 and 12. While both TNF-α and IL-1β levels were below detection limits, IL-6 could be readily measured in all samples. Secretion of IL-6 strongly increased during DSS-induced colitis and reached levels of approximately 40 pg/ml on day 10 in mice of both genotypes (Fig 6). Concomitant with the resolution of disease symptoms, IL-6 levels in GR^flox^ mice declined again, reaching almost baseline levels on day 12 (Fig 6). In contrast, IL-6 secretion in GR^lysM^ mice further increased until day 12 and was significantly higher than in GR^flox^ mice at this time point (Fig 6). Collectively, these findings indicate that differences in systemic IL-6 levels presumably contribute to the exaggerated tissue damage and worsened clinical symptoms.

### Regulation of gene expression in the colon

**Fig 7:**
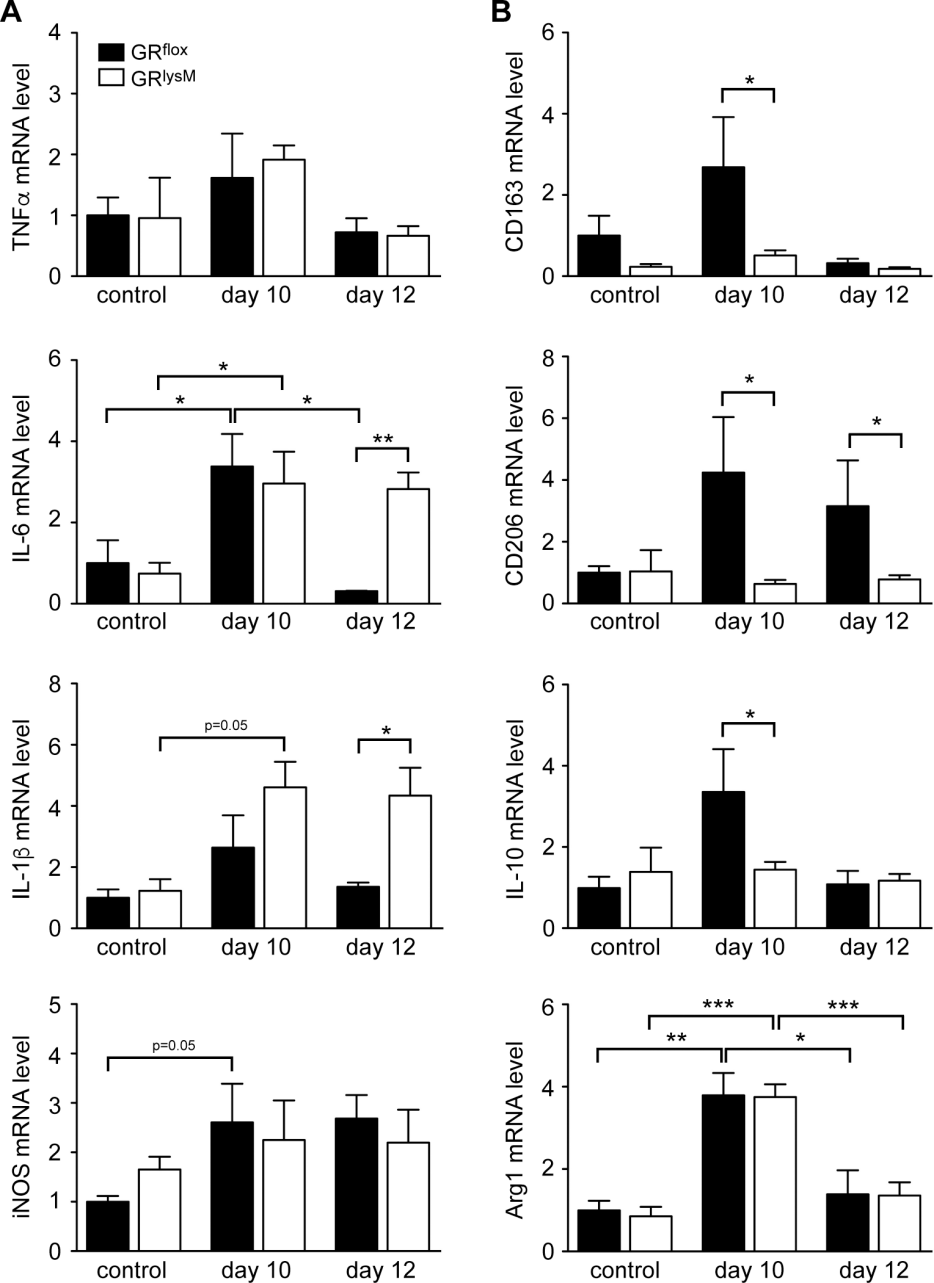
Gene expression in the colon during DSS-induced colitis in GR^flox^ and GR^lysM^ mice. Mice received 2% DSS in the drinking water for 8 days and were sacrificed on day 10 or 12. Mice receiving tap water served as controls. Colonic tissue was removed followed by mRNA isolation. Relative expression of the (A) M1 genes TNF-α, IL-6, IL-1β, and iNOS as well as the (B) M2 genes CD163, CD206, IL-10, and Arg1 was determined by quantitative RT-PCR using HPRT for normalization. Afterwards, the values were further normalized to F4/80 mRNA levels, and gene expression in untreated GR^flox^ control mice was arbitrarily set to 1. GR^flox^ mice: N = 5/5/4 (con/10/12), GR^lysM^ mice: N = 6/10/8 (con/DSS). Statistical analysis was performed by unpaired two-tailed Student’s t test (*: p <0.05, **: p <0.01, ***: p <0.001).

One of the major functions of endogenous GC is to keep inflammatory responses at bay by suppressing pro-inflammatory cytokine production in macrophages and neutrophils [15]. In parallel, GC promote the expression of scavenger receptors and anti-inflammatory cytokines [18, 29]. To explore whether the aggravated disease in GR^lysM^ mice during the resolution phase of DSS-induced colitis coincides with altered local gene expression, we performed a quantitative RT-PCR analysis. Since we had previously found that macrophages were more abundant in the colon of mutant mice, all values were corrected for F4/80 expression. TNF-α mRNA levels were largely unaltered during DSS-induced colitis but IL-6 and IL-1β mRNA levels were increased on day 10 (Fig 7A). In GR^flox^ mice, expression of these two cytokines declined again on day 12 whereas it remained significantly elevated in GR^lysM^ mice (Fig 7A). CD163, CD206 and IL-10 mRNA levels were strongly upregulated in the colon of GR^flox^ mice on day 10 but remained unaltered in GR^lysM^ mice (Fig 7B). Gene expression in GR^flox^ mice returned to control levels on day 12, with the exception of CD206 which was still elevated at this time point (Fig 7B). Surprisingly, regulation of iNOS and Arg1 was unaltered by the mutation despite their involvement in NO production (Fig 7A and 7B). Collectively, GR ablation in myeloid cells impacts macrophage polarization during DSS-induced colitis, which might contribute to the defective tissue repair in GR^lysM^ mice.

## Discussion

DSS-induced colitis is an animal model that has been shown to be especially suitable to study innate immune mechanisms in acute and chronic intestinal inflammation [20, 30]. In this study we aimed to clarify the role of the GR in myeloid cells for the modulation of IBD using this model. Firstly intestinal macrophages play an important role in the maintenance of gut homeostasis, mainly due to their unique ability to detect and phagocytose potentially harmful microorganisms without generating pro-inflammatory signals [10, 31]. Secondly GC are the standard first line therapy for the treatment of IBD [6]. In part, GC activity is related to their ability to induce an anti-inflammatory phenotype in macrophages and neutrophils [32]. However, as the GR is almost ubiquitously expressed, other cell types are influenced by GC as well, for instance intestinal epithelial cells. We therefore wanted to determine to which extent endogenous GC influence intestinal inflammation by impacting macrophage and neutrophil activity.

Our findings reported here revealed that the GR in myeloid cells has no influence on the control of DSS-induced colitis in the acute phase of the disease when tissue damage occurs and cytokine production takes place. However, during the recovery phase of the disease the control of macrophages and neutrophils by endogenous GC is critical, i.e. at the stage when DSS is withdrawn and tissue repair mechanisms are being triggered, as illustrated by the impaired recovery of disease symptoms observed in mutant mice. While the clinical and histopathological score in GR^flox^ mice declined after the peak of the disease on day 10, we did not find any relevant improvement of clinical symptoms in GR^lysM^ mice up to day 12. In contrast, weight loss and DAI score continued to increase. We therefore conclude that GR expression in myeloid cells is essential for the induction of tissue repair mechanisms after intestinal tissue damage. Of note, the clinical symptoms we observed and measured were in line with our histological evaluation of colon samples. Tissue damage characterized by crypt distortion, infiltration of inflammatory cells, and loss of goblet cells was similar in DSS-treated mice of both genotypes on day 10 and subsequently improved in GR^flox^ but not GR^lysM^ mice. In contrast, the length of the colon remained unaltered between day 10 and 12, presumably because the time span was insufficient for normalization of this characteristic feature of intestinal inflammation.

The inflammatory infiltrate in DSS-induced colitis is composed of several types of leukocytes. In line with previous publications we found that macrophages were the predominant cell type in both the healthy and the inflamed colon [26, 33]. In addition, we identified some T cells but almost no neutrophils in the colon of untreated mice. After DSS treatment, however, these cell types were recruited to the lamina propria in significant numbers as well. Interestingly, our analyses showed that macrophages were more abundant in GR^lysM^ mice than in GR^flox^ mice on day 12 of the disease. In contrast, neutrophil and T cell numbers were similar. It thus appears that GR expression in macrophages and their abundance in the colon coincide, and that this feature affects the resolution of DSS-induced colitis. However, whether GC alter the migration or rather the survival of macrophages is yet unclear.

Mice with a myeloid cell-specific GR deletion were previously shown to be highly prone to systemic inflammation due to their inability to control pro-inflammatory cytokines [16]. When analyzing systemic IL-6 secretion in DSS-induced colitis, we found a massive increase at the peak of the disease as well. While cytokine levels in GR^flox^ mice declined thereafter again, we found them to be even more increased on day 12 in GR^lysM^ mice. This finding indicates that the failure of GR^lysM^ mice to resolve the disease coincides with their inability to dampen systemic IL-6 secretion. In addition, we also analyzed the expression of several M1 and M2 genes in the colon. While we found no significant differences in TNF-α expression in colonic tissue of mice of both genotypes, IL-1β and IL-6 mRNA levels in GR^lysM^ mice were increased on day 10 and declined again thereafter in GR^flox^ mice. In contrast, both cytokines remained elevated in GR^lysM^ mice. Notably, this observation is in line with previous data indicating that the susceptibility of GR^lysM^ mice to systemic inflammation was linked to a failure to suppress IL-1β and IL-6 but not TNF-α [16]. Furthermore, expression of genes characteristic for an M2 macrophage polarization were also affected by the absence of the GR in myeloid cells. The scavenger receptors CD163 and CD206 as well as the anti-inflammatory cytokine IL-10 were selectively induced in GR^flox^ but not GR^lysM^ mice during DSS-induced colitis. It is well known that GC foster tissue repair and dampen immune responses by upregulating M2 genes [34]. In agreement with previous findings in a mouse model of multiple sclerosis [18], our results therefore suggest that dysregulation of CD163, CD206 and IL-10 impacts the resolution of organ-specific inflammation. In contrast, expression of iNOS and Arg1 was unaltered in the colon of GR^lysM^ mice, which contrasts with observations made in two models of neuroinflammation [18, 35]. Altogether, our data reveal a switch in macrophage polarization in the colon during the resolution phase of DSS-induced colitis from an M2 phenotype in GR^flox^ mice to a more M1 biased phenotype in GR^lysM^ mice, which presumably impacts colonic inflammation and tissue repair.

Despite the severe side effects that can accompany GC therapy and the fact that only 40% of all patients show an efficient long-term response, prednisolone and other GC derivatives are widely used for first line treatment of IBD, especially in patients that are refractory to the application of 5-aminosalicylates or sulfasalazines [36]. As a matter of fact, the occurrence of adverse GC effects [6] mainly relates to the circumstance that the GR is almost ubiquitously expressed [37]. Consequently, attempts to restrict GC action to specific cell types have been made by targeted application of GC using carrier systems such as liposomes, polymer-drug conjugates, inorganic-organic hybrid nanoparticles (IOH-NP), and antibody-drug conjugates (ADC) [7]. IOH-NP containing betamethasone reduced clinical symptoms in a mouse model of multiple sclerosis, an effect which exclusively depended on the modulation of macrophage function [38]. Similarly, ADC composed of an anti-CD163 antibody and dexamethasone (Dex) exhibited highly potent anti-inflammatory activity in rat models of endotoxemia and non-alcoholic steatohepatitis, which was due to the selective targeting of M2 macrophages [39, 40]. Finally, a copolymer-Dex conjugate was found to improve clinical symptoms of DSS-induced colitis whereas it did not induce osteoporosis, a major side-effect of GC therapy [8, 9]. Our finding that macrophages are important target cells of GC in IBD and the observation that these cells are suitable for drug delivery through nanoformulations therefore suggest that cell-directed application of GC might be a promising strategy to improve UC and CD therapy [41]. However, it is noteworthy that there is also evidence arguing against the targeting of GC to myeloid cells. It was reported that treatment of DSS-induced colitis using Dex-loaded liposomes aggravated the disease although others observed a beneficial effect on clinical symptoms when using free Dex [42, 43]. It will therefore be important to further analyze the efficacy of GC therapy in GR^lysM^ mice using different drug formulations, although this has to be accomplished in a chronic colitis model since GC were paradoxically found to aggravate disease symptoms in acute models such as the one employed here [43].

Taken together, our findings provide evidence that GC contribute to the recovery from intestinal inflammation by their ability to control myeloid cell abundance and activity in the colon.

## Supporting information

S1 Checklist ARRIVE Checklist.pdf. (PDF)

## References

[1] Perez-Lopez A, Behnsen J, Nuccio SP, Raffatellu M. Mucosal immunity to pathogenic intestinal bacteria. Nat Rev Immunol. 2016;16(3):135–48. doi: 10.1038/nri.2015.17 26898110 10.1038/nri.2015.17

[2] Danese S, Fiocchi C. Ulcerative colitis. N Engl J Med. 2011;365(18):1713–25. doi: 10.1056/NEJMra1102942 22047562 10.1056/NEJMra1102942

[3] Podolsky DK. Inflammatory bowel disease. N Engl J Med. 2002;347(6):417–29. doi: 10.1056/NEJMra020831 12167685 10.1056/NEJMra020831

[4] Ananthakrishnan AN. Epidemiology and risk factors for IBD. Nat Rev Gastroenterol Hepatol. 2015;12(4):205–17. doi: 10.1038/nrgastro.2015.34 25732745 10.1038/nrgastro.2015.34

[5] Shah B, Mayer L. Current status of monoclonal antibody therapy for the treatment of inflammatory bowel disease. Expert Rev Clin Immunol. 2010;6(4):607–20. doi: 10.1586/eci.10.45 20594134 10.1586/eci.10.45 PMC2939324

[6] Pithadia AB, Jain S. Treatment of inflammatory bowel disease (IBD). Pharmacol Rep. 2011;63(3):629–42. 21857074 10.1016/s1734-1140(11)70575-8

[7] Lühder F, Reichardt HM. Novel Drug Delivery Systems Tailored for Improved Administration of Glucocorticoids. Int J Mol Sci. 2017;18(9). 10.3390/ijms18091836 PMC5618485 28837059

[8] Ren K, Yuan H, Zhang Y, Wei X, Wang D. Macromolecular glucocorticoid prodrug improves the treatment of dextran sulfate sodium-induced mice ulcerative colitis. Clin Immunol. 2015;160(1):71–81. doi: 10.1016/j.clim.2015.03.027 25869296 10.1016/j.clim.2015.03.027

[9] Ren K, Dusad A, Yuan F, Yuan H, Purdue PE, Fehringer EV, et al Macromolecular prodrug of dexamethasone prevents particle-induced peri-implant osteolysis with reduced systemic side effects. J Control Release. 2014;175:1–9. doi: 10.1016/j.jconrel.2013.11.024 24326124 10.1016/j.jconrel.2013.11.024 PMC3946775

[10] Heinsbroek SE, Gordon S. The role of macrophages in inflammatory bowel diseases. Expert Rev Mol Med. 2009;11:e14 doi: 10.1017/S1462399409001069 19439108 10.1017/S1462399409001069

[11] Gordon S, Martinez FO. Alternative activation of macrophages: mechanism and functions. Immunity. 2010;32(5):593–604. doi: 10.1016/j.immuni.2010.05.007 20510870 10.1016/j.immuni.2010.05.007

[12] Reber SO, Obermeier F, Straub RH, Falk W, Neumann ID. Chronic intermittent psychosocial stress (social defeat/overcrowding) in mice increases the severity of an acute DSS-induced colitis and impairs regeneration. Endocrinology. 2006;147(10):4968–76. doi: 10.1210/en.2006-0347 16794011 10.1210/en.2006-0347

[13] Bouguen G, Langlois A, Djouina M, Branche J, Koriche D, Dewaeles E, et al Intestinal steroidogenesis controls PPARgamma expression in the colon and is impaired during ulcerative colitis. Gut. 2015;64(6):901–10. doi: 10.1136/gutjnl-2014-307618 25053717 10.1136/gutjnl-2014-307618

[14] Taves MD, Gomez-Sanchez CE, Soma KK. Extra-adrenal glucocorticoids and mineralocorticoids: evidence for local synthesis, regulation, and function. Am J Physiol Endocrinol Metab. 2011;301(1):E11–24. doi: 10.1152/ajpendo.00100.2011 21540450 10.1152/ajpendo.00100.2011 PMC3275156

[15] Tuckermann JP, Kleiman A, McPherson KG, Reichardt HM. Molecular mechanisms of glucocorticoids in the control of inflammation and lymphocyte apoptosis. Crit Rev Cl Lab Sci. 2005;42(1):71–104. 10.1080/10408360590888983 15697171

[16] Kleiman A, Hübner S, Rodriguez Parkitna JM, Neumann A, Hofer S, Weigand MA, et al Glucocorticoid receptor dimerization is required for survival in septic shock via suppression of interleukin-1 in macrophages. FASEB J. 2012;26(2):722–9. doi: 10.1096/fj.11-192112 22042221 10.1096/fj.11-192112

[17] Vettorazzi S, Bode C, Dejager L, Frappart L, Shelest E, Klaßen C, et al Glucocorticoids limit acute lung inflammation in concert with inflammatory stimuli by induction of SphK1. Nat Commun. 2015;6. 10.1038/ncomms8796 PMC4518295 26183376

[18] Schweingruber N, Haine A, Tiede K, Karabinskaya A, van den Brandt J, Wüst S, et al Liposomal encapsulation of glucocorticoids alters their mode of action in the treatment of experimental autoimmune encephalomyelitis. J Immunol. 2011;187(8):4310–8. doi: 10.4049/jimmunol.1101604 21918186 10.4049/jimmunol.1101604

[19] Wüst S, van den Brandt J, Tischner D, Kleiman A, Tuckermann JP, Gold R, et al Peripheral T cells are the therapeutic targets of glucocorticoids in experimental autoimmune encephalomyelitis. J Immunol. 2008;180(12):8434–43. 18523311 10.4049/jimmunol.180.12.8434

[20] Wirtz S, Neufert C, Weigmann B, Neurath MF. Chemically induced mouse models of intestinal inflammation. Nat Protoc. 2007;2(3):541–6. doi: 10.1038/nprot.2007.41 17406617 10.1038/nprot.2007.41

[21] AlSharari SD, Akbarali HI, Abdullah RA, Shahab O, Auttachoat W, Ferreira GA, et al Novel insights on the effect of nicotine in a murine colitis model. J Pharmacol Exp Ther. 2013;344(1):207–17. doi: 10.1124/jpet.112.198796 23115221 10.1124/jpet.112.198796 PMC3533410

[22] Whittem CG, Williams AD, Williams CS. Murine Colitis modeling using Dextran Sulfate Sodium (DSS). J Vis Exp. 2010;(35). 10.3791/1652 PMC2841571 20087313

[23] Platt AM, Bain CC, Bordon Y, Sester DP, Mowat AM. An independent subset of TLR expressing CCR2-dependent macrophages promotes colonic inflammation. J Immunol. 2010;184(12):6843–54. doi: 10.4049/jimmunol.0903987 20483766 10.4049/jimmunol.0903987

[24] Uhmann A, van den Brandt J, Dittmann K, Hess I, Dressel R, Binder C, et al T cell development critically depends on prethymic stromal patched expression. J Immunol. 2011;186(6):3383–91. doi: 10.4049/jimmunol.1001939 21317383 10.4049/jimmunol.1001939

[25] Kerr TA, Ciorba MA, Matsumoto H, Davis VR, Luo J, Kennedy S, et al Dextran sodium sulfate inhibition of real-time polymerase chain reaction amplification: a poly-A purification solution. Inflamm Bowel Dis. 2012;18(2):344–8. doi: 10.1002/ibd.21763 21618356 10.1002/ibd.21763 PMC3600644

[26] Do A, Reid RC, Lohman RJ, Sweet MJ, Fairlie DP, Iyer A. An HDAC6 Inhibitor Confers Protection and Selectively Inhibits B-Cell Infiltration in DSS-Induced Colitis in Mice. J Pharmacol Exp Ther. 2017;360(1):140–51. doi: 10.1124/jpet.116.236711 27827303 10.1124/jpet.116.236711

[27] Yan Y, Kolachala V, Dalmasso G, Nguyen H, Laroui H, Sitaraman SV, et al Temporal and spatial analysis of clinical and molecular parameters in dextran sodium sulfate induced colitis. PLoS One. 2009;4(6):e6073 doi: 10.1371/journal.pone.0006073 19562033 10.1371/journal.pone.0006073 PMC2698136

[28] Tuckermann JP, Kleiman A, Moriggl R, Spanbroek R, Neumann A, Illing A, et al Macrophages and neutrophils are the targets for immune suppression by glucocorticoids in contact allergy. J Clin Invest. 2007;117(5):1381–90. doi: 10.1172/JCI28034 17446934 10.1172/JCI28034 PMC1849982

[29] Reichardt SD, Weinhage T, Rotte A, Föller M, Oppermann M, Lühder F, et al Glucocorticoids induce gastroparesis in mice through depletion of l-arginine. Endocrinology. 2014;155(10):3899–908. doi: 10.1210/en.2014-1246 25057793 10.1210/en.2014-1246

[30] Kersting S, Behrendt V, Kersting J, Reinecke K, Hilgert C, Stricker I, et al The impact of JNK inhibitor D-JNKI-1 in a murine model of chronic colitis induced by dextran sulfate sodium. J Inflamm Res. 2013;6:71–81. doi: 10.2147/JIR.S40092 23667316 10.2147/JIR.S40092 PMC3650567

[31] Platt AM, Mowat AM. Mucosal macrophages and the regulation of immune responses in the intestine. Immunol Lett. 2008;119(1–2):22–31. doi: 10.1016/j.imlet.2008.05.009 18601952 10.1016/j.imlet.2008.05.009

[32] Lim HY, Müller N, Herold MJ, van den Brandt J, Reichardt HM. Glucocorticoids exert opposing effects on macrophage function dependent on their concentration. Immunology. 2007;122(1):47–53. doi: 10.1111/j.1365-2567.2007.02611.x 17451463 10.1111/j.1365-2567.2007.02611.x PMC2265978

[33] Stevceva L, Pavli P, Husband AJ, Doe WF. The inflammatory infiltrate in the acute stage of the dextran sulphate sodium induced colitis: B cell response differs depending on the percentage of DSS used to induce it. BMC Clin Pathol. 2001;1(1):3 doi: 10.1186/1472-6890-1-3 11580872 10.1186/1472-6890-1-3 PMC57007

[34] Martinez FO, Sica A, Mantovani A, Locati M. Macrophage activation and polarization. Front Biosci. 2008;13:453–61. 17981560 10.2741/2692

[35] Montes-Cobos E, Schweingruber N, Li X, Fischer HJ, Reichardt HM, Lühder F. Deletion of the Mineralocorticoid Receptor in Myeloid Cells Attenuates Central Nervous System Autoimmunity. Front Immunol. 2017;8:1319 doi: 10.3389/fimmu.2017.01319 29081780 10.3389/fimmu.2017.01319 PMC5645513

[36] Baumgart DC, Sandborn WJ. Inflammatory bowel disease: clinical aspects and established and evolving therapies. Lancet. 2007;369(9573):1641–57. doi: 10.1016/S0140-6736(07)60751-X 17499606 10.1016/S0140-6736(07)60751-X

[37] Cole TJ, Blendy JA, Monaghan AP, Krieglstein K, Schmid W, Aguzzi A, et al Targeted disruption of the glucocorticoid receptor gene blocks adrenergic chromaffin cell development and severely retards lung maturation. Genes Dev. 1995;9(13):1608–21. 7628695 10.1101/gad.9.13.1608

[38] Montes-Cobos E, Ring S, Fischer HJ, Heck J, Strauss J, Schwaninger M, et al Targeted delivery of glucocorticoids to macrophages in a mouse model of multiple sclerosis using inorganic-organic hybrid nanoparticles. J Control Release. 2017;245:157–69. doi: 10.1016/j.jconrel.2016.12.003 27919626 10.1016/j.jconrel.2016.12.003

[39] Graversen JH, Svendsen P, Dagnaes-Hansen F, Dal J, Anton G, Etzerodt A, et al Targeting the hemoglobin scavenger receptor CD163 in macrophages highly increases the anti-inflammatory potency of dexamethasone. Mol Ther. 2012;20(8):1550–8. doi: 10.1038/mt.2012.103 22643864 10.1038/mt.2012.103 PMC3412497

[40] Svendsen P, Graversen JH, Etzerodt A, Hager H, Roge R, Gronbaek H, et al Antibody-Directed Glucocorticoid Targeting to CD163 in M2-type Macrophages Attenuates Fructose-Induced Liver Inflammatory Changes. Mol Ther Methods Clin Dev. 2017;4:50–61. doi: 10.1016/j.omtm.2016.11.004 28344991 10.1016/j.omtm.2016.11.004 PMC5363319

[41] Ricart E, Jauregui-Amezaga A, Ordas I, Pino S, Ramirez AM, Panes J. Cell therapies for IBD: what works? Curr Drug Targets. 2013;14(12):1453–9. 24160439 10.2174/13894501113146660234

[42] Crielaard BJ, Lammers T, Morgan ME, Chaabane L, Carboni S, Greco B, et al Macrophages and liposomes in inflammatory disease: friends or foes? Int J Pharm. 2011;416(2):499–506. doi: 10.1016/j.ijpharm.2010.12.045 21238559 10.1016/j.ijpharm.2010.12.045

[43] Kojouharoff G, Hans W, Obermeier F, Mannel DN, Andus T, Scholmerich J, et al Neutralization of tumour necrosis factor (TNF) but not of IL-1 reduces inflammation in chronic dextran sulphate sodium-induced colitis in mice. Clin Exp Immunol. 1997;107(2):353–8. doi: 10.1111/j.1365-2249.1997.291-ce1184.x 9030875 10.1111/j.1365-2249.1997.291-ce1184.x PMC1904573

